# Hallucinations and related concepts—their conceptual background

**DOI:** 10.3389/fpsyg.2015.00991

**Published:** 2015-07-27

**Authors:** Diogo Telles-Correia, Ana Lúcia Moreira, João S. Gonçalves

**Affiliations:** Clinica Universitaria de Psiquiatria e Psicologia, Faculty of Medicine, University of Lisbon, Lisbon, Portugal

**Keywords:** hallucinations, pseudohallucinations, hallucinosis, illusions, psychopathology

## Abstract

Prior to the seventeenth century, the experiences we now name hallucinations were valued within a cultural context, they could bring meaning to the subject or the world. From mid-seventeenth to eighteenth centuries, they acquire a medical quality in mental and organic illnesses. However, the term was only fully integrated in psychiatry by Esquirol in the eighteenth–nineteenth centuries. By then, a controversy begins on whether hallucinations have a perceptual or intellectual origin. Esquirol favors the intellectual origin, describing them as an involuntary exercise of memory and imagination. By the twentieth century, some authors maintain that hallucinations are a form of delusion (Ey), while others describe them as a change in perception (Jaspers, Fish). More integrated perspectives like those proposed by Alonso Fernandez and Luque, highlights the heterogeneity of hallucinations and the multiplicity of their types and causes. The terms pseudohallucination, illusion, and hallucinosis are grafted into the concept of hallucination. Since its introduction the term pseudohallucination has been used with different meanings. The major characteristics that we found associated with pseudohallucinations were “lack of objectivity” and “presence of insight” (differing from hallucinations). Illusions are unanimously taken as distortions of real objects. Hallucinosis, first described in the context of alcohol consumption, is generally considered egodystonic, in which insight is preserved. These and other controversial aspects regarding the evolution of the term hallucination and all its derivative concepts are discussed in this paper.

## Introduction

Hallucination is one of the most relevant symptoms in psychiatry. It is also one of the hardest to define and delimitate from other psychopathological concepts. This latter aspect in particular led to the emergence of other related concepts like “pseudohallucination,” “illusion,” and “hallucinosis.” The etymology of the word hallucination is controversial. It may have had origin in the Latin word *allucinor, allucinaris*, used by Cicero, meaning the intent to mislead or equivocate (Corominas, 1973). It may also have originated from the Latin compound *ad lucem* (ad-*next to*; *lucem*-light; Barcia, 1903). Evidence for similar concepts may be found in Christian authors. Saint Augustine, for instance, tried to characterize visions in mystical experiences by distinguishing three meanings for the expression *videre* (latin word for “to see”): corporal (as in the visual experience of the external world through the senses), imaginative (as in the representation of images and objects that have a temporal but not spatial location), and intellectual (as in abstract concepts that lack a spatial and temporal location; Sarbin and Juhasz, 1967). Saint Thomas Aquinas established a difference between normal and false perceptions and argued that a vision (*visio*) was a natural phenomenon instigated by God or the devil (Sarbin and Juhasz, 1967).

Only after the seventeenth/eighteenth centuries have hallucinations acquired a scientific/medical sense. The term was then used to designate organic conditions (afflictions of the cornea and diplopia) and mental disorders (strange noises, premonitions, and appearances; Luque and Villagrán, 2000). According to Berrios (1996), “variously named, these experiences were in earlier times culturally integrated and semantically pregnant, i.e. their content was believed to carry a message for the individual or the world” (p. 35). With the medicalization of the term, the semantic nature was lost and hallucinations came to be considered, first, as diseases or independent syndromes and, later on, as symptoms that characterize different diseases (Berrios, 1996).

In this article we aim to review the evolution of the term “hallucination” up to present time, as well as its related concepts, such as “pseudohallucination,” “illusion,” and “hallucinosis.”

## Eighteenth and Nineteenth Centuries

Esquirol achieved the major theoretical advance on hallucinations, introducing the term to psychiatry. According to Ey (1939), Esquirol brought together psychiatry and the patient with hallucinations. Before, hallucinations were only considered in one sensory modality (vision), but Esquirol (1845) combined in his designation experiences from various sensory modalities: “Hallucinations of sight… have been denominated visions. Who would dare to say visions of hearing, visions of taste, visions of smell? … A generic name is needed. I have proposed the word hallucination” (p. 110). Furthermore, Esquirol argued that hallucinations were a form of *delirium* (“*une certaine forme de délire*”), or a symptom of *delirium* (*delirium* was then considered a synonym for madness, a syndrome that affected several psychopathological areas: thinking, perception, humor, etc.). For Esquirol (1845), “a person is said to labor under a hallucination, when they have a thorough conviction of the perception of a sensation, when no external object… has impressed the senses” (p. 94). Esquirol (1845) also noted that “this symptom of *delirium* has been mistakenly identified by all authors with local lesions of the senses” (p. 94). According to him, hallucinations were different from illusions and only the latter truly correspond to sensory errors: “In hallucinations everything happens in the brain: visionaries dream awake. The activity of the brain is so energetic that the visionary or the hallucinated gives a body and reality to images and ideas that memory reproduces, without the intervention of the senses. In illusions, on the contrary, the sensibility of the nerve extremities is altered” (Esquirol, 1845, p. 111). According to Esquirol (1845), every type of hallucination was based on the same pathophysiology: “the images, ideas and notions which seem to belong to the function alteration of these free senses (hearing, taste, smell) … are produced by the same causes” (p. 110). By this time, the hallucinations were often referred to as “perceptions devoid of an object,” a definition often erroneously associated with Esquirol. On the contrary, Esquirol always maintained that the hallucination was not a perception but a “form of *delirium* that makes patients believe they have a perception,” meaning, the conviction of having a perception but not actually having a perception. In fact, Falret (1864) states that “the hallucination is a perception without object, as has been often repeated” (p. 264).

The work of Esquirol emerged at a stage when there was a controversy about hallucinations in French Psychiatry, a debate which revolved around two fundamental dichotomies: (1) If hallucinations arise simply by an “involuntary exercise of memory and imagination”—as advocated by Esquirol and others—or, rather, are the result of a sensory abnormality (central or peripheral); (2) If hallucinations were always pathological and, therefore, only occurring in the context of mental disease or whether they could occur without mental disease. This last question arises from the first, and goes back to the self-report of two patients suffering from these symptoms.

**FIGURE 1:**
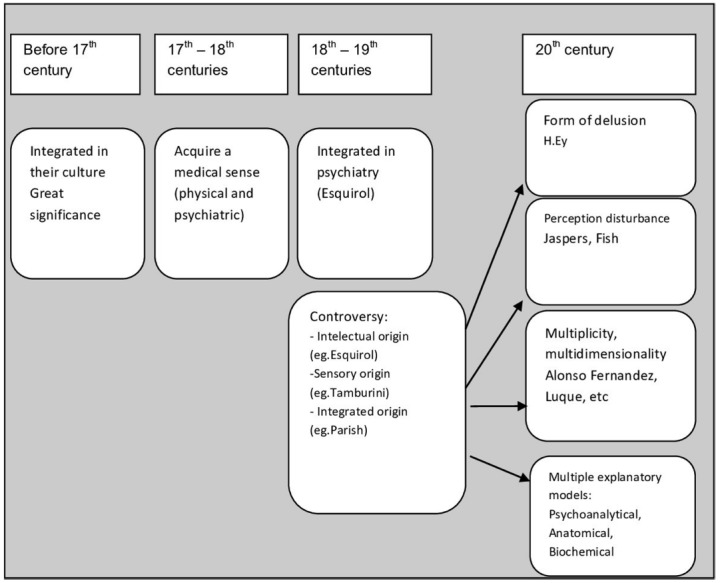
**Prior to the seventeenth century, the experiences we now name hallucinations were valued within a cultural context, they could bring meaning to the subject or the world.** From mid-seventeenth and eighteenth centuries, they acquire a medical quality to designate mental and organic illnesses. However, the term is fully integrated in psychiatry by Esquirol in the eighteenth-nineteenth centuries. By then, a controversy begins on whether hallucinations have a perceptual or intellectual origin. Esquirol favors the intellectual origin (involuntary exercise of memory and imagination). Tamburini believes they are the result of the stimulation of the centers of imaging formation in the brain, but Parish advances with a more integrative approach. In the twentieth century, some authors maintain that hallucinations are a form of delusion (Ey), while others describe it as a change in perception (Jaspers, Fish). A more integrated perspective, like the one proposed by Alonso Fernandez and Luque, highlights the heterogeneity of hallucinations and the multiplicity of their types and causes, even within hallucinations with a psychiatric (and non-organic) cause. Multiple explanatory models were also developed during the twentieth century.

Nicolai published in 1799 an essay called “*Memory of the Apparition of Ghosts or Spectres Caused by Disease with Psychological Considerations*” (Ferriar, 1813; Brierre de Boismont, 1862). In this work, Nicolai described his own visual hallucinatory experiences, where insight had been preserved. This case was included by Brierre of Boismont in the category of “hallucinations compatible with reason” (Berrios, 1996). In his book “*Les Farfadets, ou Tous les démons ne sont pas de l’autre monde*” (published in 1821) Berbiguier reported experiences compatible with hallucinations and delusional ideas, where insight was not preserved. This case was considered paradigmatic of pathological hallucinations (Berrios, 1996). Baillarger, in 1844, wrote an essay (for a contest of the Royal Academy of Medicine), in which he classified hallucinations in its several modalities (claiming that the auditory hallucinations were “the most frequent,” although visual hallucinations were “easier to study and understand”) and tried to respond to the dispute in which the psychological or psycho-sensorial origin of hallucinations was questioned. In this context Baillarger (1886) proposed two types of hallucinations: psycho-sensorial (arising from a combination of the action of imagination and the sensory organs) and psychological (independent of the sense organs). Falret (1864), who found Baillarger’s division unhelpful regarding clinical practice, defended Esquirol’s strictly intellectual perspective of hallucinations: “the hallucination demands the intervention of the intellectual faculties, the memory and the imagination… it is a phenomenon with a psychic nature and not a sensory nature” (p. 23). Michéa (1851) in its “*Du Delire Des Sensations*,” from 1846, also considers hallucinations as a transformation—usually involuntary—of memory and imagination into something that resembles a sense-perception. Griesinger (1867) defines hallucinations as “subjective images that are projected externally and acquire an apparent objectivity and reality” (p. 86). Tamburini (1881) questioned the accepted view (particularly defended by the French school) that proposed the origin of hallucinations to be predominantly psychological. According to Tamburini (1881) all hallucinations were a result of excitation of the centers of formation of images and other sensations in the brain. Several authors of this time, such as Chaslin (1912), accepted this position and developed it. This unitary vision of hallucinations promoted a divorce between history and the social context of the patient and the content of the hallucinations, which was caused by random stimulation of the nerve centers. According to this view, even the presence or absence of insight was determined by the intensity of stimulation of those centers (Berrios, 1996) (Figure 1).

Edmund Parish von Senftenberg (1861–1916), a German researcher, published, in 1894, a seminal work in this area, “*Uber die Trugwahrnehmung”* (“*Hallucinations and Illusions*”; Parish, 1897). Here, the author suggests that the term “misleading perceptions” could encompass all concepts related to hallucination and illusion: “all hallucinations and illusions can be designated by misleading perceptions, whether observed in the sane or the insane, whether occurring in sleep or in the waking state, whether arising spontaneously or experimentally induced” (Parish, 1897, p. 17). Parish puts forward a new solution, conceptualized as the theory of “associative pathways” and that included the afferent and efferent pathways of the brain. According to him, in a normal perception there is information originated by the peripheral sensory organs and transmitted to the brain, affecting region A, which in turn communicates with other regions B, C, etc. From these regions efferent information is created, which is then, transmitted to the sensory organs a, b, c, etc. Thus, normal perception arises. Conversely, on occasion, after zones A, B, C, being excited, efferent information is locked in its usual channels, and has to find different ways to reach the sensory organs, thus creating a new perception: the hallucination. In other cases, a given perception stimulates region A, but cannot stimulate B and C, resulting in an incomplete efferent information (for instance only a). In this case, illusion arises. On the other hand, Parish (1897) states that “the great controversy on whether hallucinations arise from sensory or ideational centres is meaningless since the sensory and imaginative centres are not separate and occupy close parts of the brain” (p. 134). In this theory, Parish overcomes extremist views and finds an integrative and original solution accepting the idea that central and peripheral sensory regions can be affected and that both the ideational and sensory regions of the brain are affected (Figure 1).

## Twentieth Century

In turn, Jaspers defines hallucinations as perceptions of morbid origin (*sinnesvorgange*), which did not appear by transformation of real perceptions (as illusions do). Citing the work of Kandinsky, he says that, unlike pseudohallucinations, hallucinations have sensory consistency (objectivity, detail and corporality) and originate outside the subject’s mind. Jaspers (1963) only intends to describe these psychopathological phenomena (as is characteristic of its descriptive psychopathology), without having the ambition to explain the pathophysiology (Figure 1).

Later, Ey defined hallucinations as a psychosensory disruption different from illusion and delusional interpretations (which for him was synonymous to the German term of delusional perception), consisting characteristically of a perception without an object (Ey et al., 1978). The three major conditions associated to hallucinations were: (1) sensory appearance of the experience; (2) conviction of its reality; (3) absence of a real object (Ey, 1973). On the other hand, he also insisted that hallucinations “are secondary to false beliefs or convictions” (Ey, 1973, p. 45), approaching the original definition by Esquirol, for whom the hallucination was a form of delusion that led patients to believe that they had a perception. This author, like other authors with a phenomenological orientation (Mayer-Gross, Mattussek, Zutt, etc), defends a unifying vision hallucination-delirium that meets in the psychotic patient with a deformation of the global reality (Castilla del Pino, 1984). He divides hallucinations into two major groups: (1) secondary to loss of structure of the field of consciousness (altered consciousness of the oneiroid type); (2) secondary to disruption of the self (present in schizophrenia; Ey, 1973). Cabalero Goas (1918–1977) also understood the hallucination as a disturbance in the distinction between the outer and inner world. To this author hallucinations have several features among which are highlighted: (1) a false perception, where falsity arises from not corresponding to any real object, and that occurs along with real perceptions; (2) a “new” phenomenon to the patient with a nature of perception that presents itself as being strange to his personality, coming from outside of the subject; (3) possession of an irresistible ability to convince the patient of its reality, to paraphrase Bumke (1946) who stated that “the power that these perceptions have over patients is higher than that of normal perceptions”; (4) aesthetic-spatial nature; (5) presentation of corporeality and its location outside the subject’s mind; (6) disintegration of the real and replacement by a quasi-reality (this concept is imported from Merleau Ponty); (7) expression of an inner psychological experience in the form of a psychosensorial manifestation; (8) existence in conjunction with a disturbance of the mental activity characterized by a loss of insight, which causes this phenomenon to be accepted as a normal sensory perception (Goas, 1966). These features underline the fact that hallucinations are a very heterogeneous group of phenomena. They appear in key groups: (1) Schizophrenia, in which they arise by loss of consciousness of the ego activity, the patient feeling totally influenced, directed and governed by the external world, with a rupture or excision of the internal unit of the perceptual world in relation to the surrounding world. In this situation, the subjective and the objective are confused, thoughts that are determined by a delusion are verbalized and are heard as coming from the outside; (2) In another group, the acute confusional syndromes, where there is a dissolution of consciousness to an oneiroid level, with a characteristically associated obnubilation; (3) In another, acute psychosis (“*bouffée délirante*”), where there is also a characteristic dissolution of consciousness to the oneiroid level, with a predominance of visual hallucinations and a very marked distress (Goas, 1966).

Lopez Ibor (1906–1991) advances with a new pathophysiology for the hallucination. According to the author, in normal perception of real objects a transmission of a signal occurs that will be deposited in the CNS (central nervous system) in the form of an engram. In the hallucination there is an abnormal activation of these engrams by direct stimulus made by the center of ideas (with their affective, intuitive component), so that individuals will experience the phenomenon with all the characteristics of a normal sensory perception. In such cases, there is a clear fusion of the inner and external worlds, both objective and subjective (Lopez Ibor, 1964).

Slade and Bentall (1988) define hallucination as any experience similar to a perception that: (1) occurs in the absence of an appropriate stimulus; (2) has all the strength and impact of the corresponding real perception; (3) is not susceptible of being voluntarily directed or controlled by those who experience it. These authors suggested that hallucinations occur as a result of the inability of the subject to distinguish if an object is real or if it is a product of the imagination, i.e., hallucinations would be caused by a deficit in the metacognitive capacity of assessment/discrimination of reality (Slade and Bentall, 1988).

Based on a thorough review of the history of the concept, Berrios (1995) recently introduced a broader classification of hallucinations, defining them as “verbal reports of sensory experiences with or without insight, not vouchsafed by a relevant stimulus” (p. 229). By doing so, Berrios leaves little room to the development of related concepts such as “pseudohallucination” and “hallucinosis,” whose theoretical validity is very debatable, according to this author.

The concept of hallucinations was also addressed within the phenomenological philosophy. The most representative example was given by Merleau-Ponty. He considers that the malfunctioning of both the power of summoning and perceptual faith originate hallucinations. The first is the capacity to bring appearances to existence. The second relates to promptings one extracts from the world. Individuals may perceive the world in an infinite number of options. Until further experience there is room for illusion. In that sense, one needs faith in order to perceive the horizons as satisfiable, as real. When both perceptual capacities mentioned are altered hallucination arises (Merleau-Ponty, 2002; Romdenh-Romluc, 2007).

Beyond descriptive psychopathology and phenomenological models of hallucinations there were many explanatory models developed throughout the twentieth century, some of them are still in investigation. One of these models was the psychoanalytical model. Freud initially believed that hallucinations resulted from forgotten traumatic experiences from childhood, which returned and forced themselves into consciousness (Bloom, 2010). He revised his theory afterwards and argued that hallucinations were fantasies or wish fulfilments, recreating things which have been lost or destroyed earlier (Eigen, 2005). For Freud “wishing ends with hallucination” (Eigen, 2005, p. 41).

Jung argued for a need to focus on the psychology of hallucinations and their content. He reported that “hallucinations contain a germ of meaning” and that “a personality, a life history, a pattern of hopes and desires” lie behind such experiences (Jung, 1963, p. 127).

On the other hand, Lacan focused on the subjective effect of hallucinations on the person that experiences them. According to Lacan “hallucination is a perceptum that has a paradoxical effect on the percipiens. Rather than a perceptum without an object, it is a perceptum that disrupts the subject. Lacan first emphasises the effects of perplexity that hallucinations evoke, in terms of disrupted signification” (Vanheule, 2011, p. 102). Later on he elaborates on this viewpoint and argues the possibility of hallucinations having a stabilizing and pacifying effect (Lacan, 1993; Vanheule, 2011).

Other models are the cognitive-perceptual, the anatomical, and the biochemical ones. Regarding cognitive-perceptual theories developed since the twentieth century, hallucinations can be seen as erroneous perception or “sensory deceptions.” Both bottom-up (or data-driven) and top-down (or conceptual) models have been distinguished. The first occurs with sensory perception impairments, for instance, when older or sensory deprived people present with auditory hallucinations, or when people with deficits in processing visual stimuli present with visual hallucinations. The second, that has been chosen by several authors, occurs in the brain of the perceiver and is not related to the external world. For Bentall (1990) hallucinations are the result of the failure of source monitoring, which is a metacognitive skill necessary to discriminate between both internal and external sources of information. For (Hoffman and McGlashan, 2006), who uses a psycholinguistic approach, a parasitic memory can disrupt language production and thus originate auditory hallucinations. For David (1994) inner speech may be at the basis of verbal hallucinations through a disruption of the “inner-voice-inner-ear system.” Other authors consider a misattribution of internal events to an external source. In this sense, hallucinations could be related to “normal intrusive thoughts” externalized due to motivational factors (Morrison et al., 1995; Kumar et al., 2009).

Several functional and structural neuroimaging studies have been preformed to try to find the anatomical correlates of hallucinations [mainly in auditory verbal hallucinations (AVH)]. The most consistent findings are an association between AVH and: (1) structural abnormalities in the Superior Temporal Gyrus and Inferior Frontal Gyrus; (2) an association between the hyperconnectivity in the Arcuate Fasciculus, (3) functional activation in Superior Temporal Gyrus and Inferior Frontal Gyrus, insula, cingulate, cerebellum, and supramarginal gyrus (McCarthy-Jones, 2012). Nevertheless, most of these studies are done in patients with many additional symptoms other than hallucinations (usually in patients with schizophrenia) and therefore these changes might not be specific to hallucinations.

A biological model based on neurotransmitters has also been proposed. This model has been developed after the emergence of antipsychotics. In schizophrenia there is evidence that very high levels of dopamine in the limbic system play a major role in the emergence of hallucinations and delusions. Antipsychotic medications, which block central dopamine activity, alleviate hallucinations in psychosis (Kapur, 2003). These medications do not only improve hallucinations but also other psychotic symptoms such as delusions. Again this model might not be specific to hallucinations.

## Pseudohallucinations

The term “pseudohallucination” (literally, false hallucination) has been used with different meanings throughout the history of its existence. The term appears to have been introduced in 1868 by Hagen, to define “illusions or sensory errors,” such as hypnagogic hallucinations (that occur in normal subjects when falling asleep and are experienced passively, unintentionally, with a lack of clarity and objectivity and maintaining awareness of their falseness; Hagen, 1868). But before Hagen, other authors have described similar phenomena without designating them with this term. On the other hand, the development of the concept pseudohallucination occurs close to the term hallucination and has even been used to respond to many of the conceptual issues that arose in relation to the latter (normality vs. abnormality, if they come from inside or outside the subject’s mind). Although the importance of distinguishing between complete perceptual experiences and pseudoperceptions had been stressed ever since the medieval christian authors (like Saint Augustine and Saint Thomas Aquinas), the first real contributions to the development of the term pseudohallucination emerged in France in the mid-nineteenth century.

By defining the concept of hallucination as a “conviction of perceiving a sense to which there is no external object,” Esquirol gives way to the possibility of the arousal of pseudohallucinatory experiences in which the patient’s conviction is significantly weaker (Esquirol, 1845).

**FIGURE 2:**
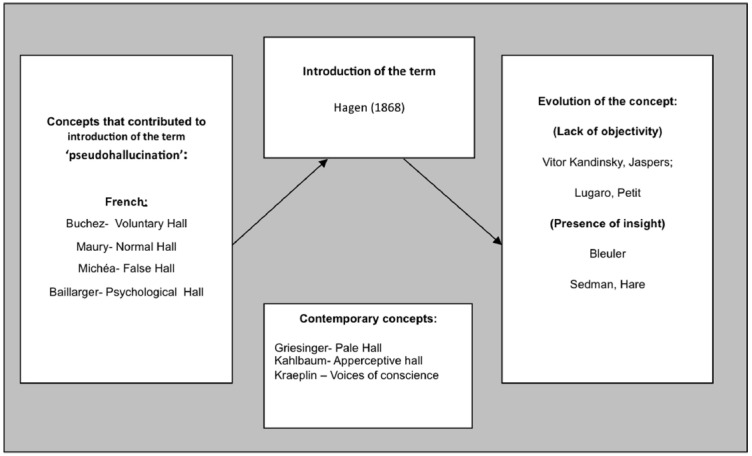
**HALL-Hallucination.** Here are represented the French concepts that could have influenced the introduction of the term “pseudohallucination” by Hagen (1868); the contemporary German concepts that are very similar to pseudohallucinations but have different names; and the further evolution of the term: some authors emphasize the lack of objectivity as the main difference between hallucinations and pseudo hallucinations (Kandinsky, Jaspers, Lugaro, Petit) but others focus instead on the presence of insight (as Bleuler, Sedman, Hare).

Other French authors also contributed to the construction of the concept of pseudohallucination, introducing some constructs that have much in common to the later concept of pseudohallucination. Buchez proposed that hallucinations should be divided between involuntary and voluntary (the latter considered normal, occurring in artists; Berrios, 1996). Maury, on the other hand, stated that hallucinations observed in mystics, usually in the context of exhaustion and prolonged fasting, correspond to the “normal hallucination type” and Boismont introduced the term “physiological hallucinations” to include the hallucinations of mystics and other visionaries whom he refuses to consider alienated (Berrios, 1996). Michéa, in 1840, referred to a type of phenomenon he called “false hallucinations,” which he considers as intermediates between an idea and true hallucinations: “False hallucination is more than an idea, since its object reveals a vivid and defined shape, which is very close to the appearance of a physical element, but is less than a true hallucination, as it will never impose itself as a real perception, however vivid and defined it is” (Michéa, 1851, pp. 113–114) (Figure 2).

Baillarger distinguishes between psychological hallucinations (depending on imagination) and psycho-sensory hallucinations (depending on both imagination and senses). He said about psychological hallucinations: “their voices are intellectual—they occur within the soul,” and that the “patients hear their thought by means of a sixth sense” (Baillarger, 1886, pp. 384–389). These statements are very close to the actual meaning of pseudohallucinations.

Nevertheless, the origin of the term pseudohallucination has been attributed to Hagen, as stated before. It was in his 1868 book “Zur Theorie der Hallucination,” that he defined them as “illusions or sensory errors,” as mentioned above, and that they could be confused with true hallucinations. In this work Hagen cites several authors such as Esquirol, Falret, Bailarger, Boismont, among others, that had described concepts close to pseudohallucinations before him. Therefore, it is not fair to attribute the origin of pseudohallucinations solely to Hagen (1868), and to forget the important contribution of other authors, namely from the French School (as apparently did Jaspers) (Figure 2).

Other Hagen contemporary authors from the German school also played a key role in developing the concept of pseudohallucination. Griesinger (1861) stated that “there is a difference between hallucination and the inner exaltation of the imagination (…) we can ask ourselves whether this difference is specific or just a matter of degree (…) I’ve seen an interesting transformation of obscure hallucinations, pale, internal (*blassen Mithallucinirens der inneren Sinne*), accompanying the perception of hallucinations with a real objective clarity” (p. 91).

Kahlbaum also published an important work on the pathophysiology and the clinic of hallucinations. According to him, there would be an organ of apperception that generated centrifugal hallucinations (or apperceptive hallucinations–closely related to the spontaneous activity of memory and little with true sensory content; Lange, 1900).

In addition to hearing clear hallucinations as voices from the outside world (which as he stated were predominant) Kraeplin (1919) noted that patients with *dementia praecox*, also sensed “voices of conscience,” “false voices,” “voices that do not speak with words,” where there is an “inner feeling in the soul.” These descriptions aligned with those corresponding to pseudohallucinations.

The term “pseudohallucination” was then further explored by other authors such as the Russian psychiatrist Kandinsky (1885) In his work, he described his hallucinatory experiences defining pseudohallucinations as “subjective perceptions similar to hallucinations, with respect to its character and vividness, but that differ from those because these do not have objective reality” (Kandinsky, 1885, p. 134) (Figure 2).

It was based on the descriptions of Kandinsky that Jaspers developed his theory about pseudohallucinations, which is, still today, the most widespread in the scientific community. According to this author pseudohallucination is a false hallucination, and a phenomenon that could be, at first sight, mistaken for a true hallucination. In pseudohallucinations there is a lack of sensory consistency (objectivity, detail, and corporality) and they are not located outside the subject’s mind. Other features of pseudohallucinations correspond to the fact that they do not usually persist for long over time and can be modified by will (initiated or interrupted; Jaspers, 1963).

Bleuler (1934) has defined pseudohallucinations in a slightly different way from Jaspers, describing them as perceptions with full sensory clarity and normal localization, but whose falsity is recognized, thus emphasizing the importance of insight.

Lugaro, a Florentine alienist, published, in 1903, a paper on pseudohallucinations in which he defines them in a very broad and current way: (1) pure representations, without the objectivity of hallucinations; (2) they present ego-dystonia (*carattere di estraneità alla personalità*); (3) they result from an irritation in the associative centers (and not in the sensory centers); (4) they may produce secondary delusions; (5) they can be seen in prolonged psychotic states as in chronic schizophrenia (Lugaro, 1903).

Petit (1913) writes *Essai sur une varieté de pseudohallucinations: les auto-représentations aperceptives*. According to him this kind of phenomena, whose conceptualization dated back to Baillarger, Kandinsky, among others, could acquire multiple presentations and had only in common that they looked very much like hallucinations. Among the various subtypes of pseudohallucinations, Petit (1913) described a group of automatic phenomena (i.e., which arise spontaneously and impose their presence on the subject), that are recognized as not being caused by any sensory source, that lack the attributes of external perceptions, and that are directly experienced in consciousness, while also being perceived as egodystonic (*créations exogenes, étrangéres par leur origine à son Moi conscient et créateur*).

It was only in the second half of the twentieth century that the Anglo-Saxon school also took its part in the conceptual development of pseudohallucinations. Sedman (1966) considered them a kind of hallucination perceived through the senses but recognized as false. Hare (1973), defined them as subjective sensory experiences of morbid origin that are not interpreted in a morbid way because they are recognized by the patient as real (Hare, 1973). Hare was thinking of classic authors, such as Bleuler, who called attention to the importance of insight, something that cannot be present in true hallucinations (Figure 2).

In DSM-IV, pseudohallucinations are mentioned in the section on Conversion Disorders and defined as a possible symptom of conversion disorder which occurs with preservation of insight in the absence of other psychotic symptoms, often involving more than one sensory modality, and frequently having a naive, childish or fantastic content and a psychological meaning (American Psychiatric Association, 2000). In DSM-5 this definition faded out, and there is no reference to the term (American Psychiatric Association, 2013).

## Illusion

The term *illusion* also appears to have been originally coined by Cicero from the Latin *illusio*, *illusionis* (deception), *illudere* (to deceive).

Esquirol called illusions “sensorial errors” and defined them as perceptions derived from sensory stimulation that are distorted by certain ideas or passions. Ever since then, most authors find consensus in defining illusion as a perceptual distortion of a real stimulus.

Ey et al. (1978) defines illusions as “falsifications of the perception of real objects” and Bleuler (1934) as “pathologically altered real perceptions.” Jaspers (1963) also incorporates Esquirol’s original concept, defining illusion as experiences that correspond to transpositions (or distortions) of real perceptions where external sensory stimuli unite with certain transposing (or distorting) elements so that in the end we cannot differentiate one from the other (Jaspers, 1963). He discriminates three types of illusions: those that stemmed from inattention, those from altered affective states, and finally “pareidolias.” Fish followed Jaspers’ view and defined illusions as stimuli from a perceived object combined with a mental image that produces a false perception (Fish, 1967).

In DSM-IV, illusion is defined as misinterpretation or misperception of a real external stimulus, and DSM-5 keeps this definition (American Psychiatric Association, 2000, 2013).

## Hallucinosis

This term was introduced by Wernicke (1906)—Alkoholhallu-zinose, alcoholic hallucinosis—referring to the presence of vivid and threatening acoustic hallucinations in excessive alcohol consumers who maintained insight and showed no disturbance of consciousness associated (Telles-Correia et al., 2014). Later on, Ey et al. (1978) defined “eidolie hallucinosique” as an hallucinatory setting with no associated delusions in patients with insight and that were egodystonic regarding the non-real characteristics of the hallucinations (the hallucinatory experience is disintegrated from the patient’s personality).

In the ICD-10, “organic hallucinosis” is defined as: “A disorder of persistent or recurrent hallucinations, usually visual or auditory, that occur in clear consciousness and may or may not be recognized by the subject as such. Delusional elaboration of the hallucinations may occur, but delusions do not dominate the clinical picture; insight may be preserved” (World Health Organization, 1992). There is no reference to the term in both the DSM-IV and DSM-5 editions (contrary to previous editions, for instance, DSM-III).

Throughout the twentieth century, the term hallucinosis was progressively attributed to other types of psycho-organic syndromes and substance-abuse associated behavioral disturbances, other than alcohol consumption.

## Conclusion

We conclude that there has been a major difficulty in both defining and limiting the concept of hallucination, ever since its first appearance; and this is likely because it belongs to a heterogeneous group of symptoms that might be found in a variety of psychiatric disorders and normal physiological states as well (as in hypnagogic hallucinations). On the other hand, there is no cause that necessarily produces a hallucination. This justifies Alonso Fernandez’s (1968) statement that “the fact that psychic phenomena like hallucinations don’t possess an intrinsic unity results in an impossibility of attributing a globally valid definition to them” (p. 501).

The types of classification of hallucinations also ranged widely throughout history, including etiological (psychic or psychosensory), nosological: (normal/pathological), and phenomenological (with or without insight, with or without sensory consistency) definitions.

If defining hallucination poses such a difficult task one can argue that defining its dependent concepts is an even bigger challenge. For instance, there is a great deal of confusion between the meanings of pseudohallucination and hallucinosis. Luque calls our attention to the fact that hallucinations, as well as delusions, should not be considered one-dimensional but, instead, as the expression of a series of dimensions or factors that constitute them (Luque and Villagrán, 2000). Chen and Berrios have isolated eleven dimensions of hallucinations in a clinically applicable scale: insight, vividness (perceptive detail), complexity, localization (spatial origin attribution), intensity, voluntary control, constancy, bizarreness, situation, attribution (toward a specific event) and connection with delusions (Chen and Berrios, 1996). Haddock conceived a scale to measure dimensions of hallucinations and delusions. The hallucination section was divided into four dimensions (emotional characteristics—negative content, impact; physical characteristics—duration, frequency; control; and cognitive interpretation; Haddock et al., 1999).

Beyond descriptive psychopathology and phenomenological models of hallucinations there were many explanatory models developed throughout the twentieth century, some of them are still in investigation. The anatomical and biochemical models are perhaps the most recent ones and that have not yet found a specific pattern for hallucinations.

There is still a lot to be clarified regarding hallucination as a psychopathological phenomenon and all its derivative concepts. The problem does not lie in its description or classification in terms of descriptive psychopathology.

The problem lies in clarifying in what the hallucination generator structure consists and if it exists as a unitarian or a multiple etiology. This is probably the major issue that supports the differences between distinct authors opinions regarding the concept of hallucination. The fact that the mentally ill patients consider it an image derived from the external world, reaching them by means of a perceptive organ, allows the hallucinatory phenomenon to be classified as a psychosensory disturbance, as it unanimously happens in current days.

Nevertheless, presently, there is no clear notion about pathophysiological mechanisms underlying the phenomenon. Therefore, the descriptive component remains essential, even though, sometimes, blinded by a fierce nosological ambition. In order to reach real biological correlates it is imperative that we go back to fundamental psychopathological symptoms and use them as a primary basis in all investigations.

Studying the history of psychopathology is a powerful way of calibration, by which language in Psychiatry can be improved and prepared for more rigorous quantification.

As Berrios (2011) postulated, the epistemology of psychopathology “has to include a combination of methods as history, philosophy and empirical investigation” (p. 39). The history of psychiatry and psychopathology brings to us some information about the social processes where concepts have evolved, philosophy clarifies if the language used is sufficiently powerful, and empirical investigation tests the validity of the new concepts toward reality (Berrios, 2011).

### Conflict of Interest Statement

The authors declare that the research was conducted in the absence of any commercial or financial relationships that could be construed as a potential conflict of interest.
